# Effect of β2-microglobulin in evaluating the severity and prognosis of brain injury: a clinical study

**DOI:** 10.1186/s12883-022-02850-8

**Published:** 2022-09-01

**Authors:** Qiqi Huo, Wenshuo Dong, Yu Gao, Yi Zhang, Xuehua Liu, Lu Yang, Ding Nan, Jing Yang

**Affiliations:** 1 Department of Neurology, Beijing Chao-Yang Hospital, Capital Medical University, Beijing, 100020 China; 2 Department of Hyperbaric Oxygen Medicine, Beijing Luhe Hospital, Capital Medical University, Beijing, 101149 China; 3 Department of Hyperbaric Oxygen Medicine, Beijing Chao-Yang Hospital, Capital Medical University, Beijing, 100020 China

**Keywords:** β2-microglobulin, Brain injury, Hyperbaric oxygen therapy

## Abstract

**Background:**

β2-microglobulin has been showing to be vital that associated with brain function and neurological diseases. This study aimed to explore the expressions of β2-microglobulin in blood and urine of the patients with brain injury, and the effect of hyperbaric oxygen therapy on the content of β2-microglobulin.

**Methods:**

This prospective study included 54 patients with brain injury and 11 healthy controls. The patients were further assigned to two groups: the conscious disturbance group (*n* = 32) and the non-conscious disturbance group (*n* = 22) depending on the Glasgow Coma Scale (GCS). The patients received routine treatment and two courses of hyperbaric oxygen therapy (2.0ATA, 60 min, once a day, 10 days for a course). In the brain injury group, blood β2-microglobulin (β2MG) and urine β2-microglobulin (β2MU) were detected respectively before and after hyperbaric oxygen therapy (HBOT). Consciousness and cognitive scores were performed, correspondingly.

**Results:**

Compared with those of the control group, levels of β2MG and β2MU in the brain injury group were significantly increased before HBOT (*P* < 0.05). Whether it was before or after HBOT, β2MG’s content in the conscious disturbance group was higher than that in the non-conscious disturbance group, while β2MU’s content was obviously higher than that of the non-conscious disturbance group only before HBOT (*P* < 0.05). Besides, the β2MU’s content in the conscious disturbance group was negatively correlated with GCS score (*R* = -0.351, *P* < 0.05) and β2MG’s content in the non-conscious disturbance group was positively correlated with the MMSE score grade (*R* = 0.598, *P* < 0.05). The ROC curve was used to assess the evaluation of β2MG and β2MU for patients with impaired consciousness with the area under the curve (AUC) of β2MG and β2MU were 0.775 and 0.796, respectively.

**Conclusion:**

The concentrations of blood β2-microglobulin and urinary β2-microglobulin were significantly increased in patients with brain injury. The concentrations of β2-microglobulin were correlated with the degree of consciousness and cognitive function. The changes tendency of β2-microglobulin may be considered as clinical monitoring index to evaluate the patient’s disturbance of consciousness and cognitive degree, and provide a basis for early assessment of prognosis.

## Introduction

β2-microglobulin is a low molecular weight protein which is produced by lymphocytes, platelets, and multinucleated leukocytes. It was separated from the urine of patients with renal tubular disease for the first time in 1968 by Berggard [1] et al. β2-microglobulin is an important part of the major histocompatibility complex I (*MHC*-I) and plays a significantly important role in antigen expression, immunoglobulin transport, iron metabolism and so on. Under the physiological condition, β2MG, producing at a constant rate, can pass through the glomerular filtration membrane easily, while 99.9% of the filtered β2MG can be reabsorbed by renal tubules in the form of pinocytosis, and are discharged with urine sightly. Therefore, the concentration of β2-microglobulin in serum is relatively stable. A study has explored that [2] β2MG is related to inflammation and arteriosclerosis and plays a significant part in the initiation of inflammation. When inflammation occurs, a large number of T lymphocytes secrete so that the production and release of β2MG rise [3, 4]. With the increase of the degree of immune stress, the level of β2MG goes up gradually, which is consistent with the level of inflammatory factors in vivo. The enhancement of inflammatory response also indicates the stress state of the immune system. Therefore, it can be observed that the level of β2MG increase in pathological conditions, such as autoimmune diseases, kidney diseases and nervous system diseases and so on.

Brain injury caused by brain diseases such as stroke and craniocerebral trauma is a common clinical disease, mainly lead to neurological damage. The high fatality rate and disability rate of brain injury seriously threaten people’s quality of life and bring a huge burden to families and society. According to the data from the Screening and Intervention Project for People at High Risk of Stroke, it is estimated that the number of stroke patients aged 40 and over in our country has reached 12.42 million [5]. Craniocerebral trauma is an important global public health problem. And the annual incidence rate in China was 313 per 100,000 [6]. The high mortality and disability rate of brain injury seriously threaten people’s quality of life and bring a huge burden to the family and society. Timely and accurate diagnosis and treatment are very important for the prognosis of the disease.

In recent years, several studies have found that β2MG has a huge influence on immune and neuroregulatory functions. It can change the development and cognitive function of the brain by regulating nerve regeneration and synaptic plasticity [7]. Dominici et al. [8] found that the degree of cognitive impairment was positively correlated with the concentrations of β2-microglobulin. What’s more, it was found that β2MG could lead to cognitive impairment by influencing the characteristic of the hippocampal neural progenitor cells (NPC) directly, such as the self-renewal, proliferation and neuronal differentiation [9, 10]. At the same time, β2MG is considered as the initiator of inflammation, its chemotaxis effect on mononuclear macrophages secreting thrombosis and tissue division factors can accelerate thrombosis and participant in the occurrence and development of atherosclerosis. Amighi et al. [11] conducted a 3-year follow-up study of 1286 patients with carotid plaque without neurological symptoms, in which 359 major adverse cardiovascular events were significantly independently associated with the increase of β2MG which was more sensitive than hypersensitive C-reactive protein. A study on women’s health by Rist et al. [12] found that a 30% increase in β2MG content would increase the risk of stroke by 18%, and higher β2MG level was correlated with the risk of ischemic stroke.

By detecting the concentration and change trend of β2-microglobulin in serum and urine, this study aims to make a preliminarily judge to the severity and development of brain injury and provide a relevant guidance for the treatment of patients.

## Patients and methods

### Clinical data

A total of 54 Chinese patients with brain injury were admitted to hospital (Beijing Chao-Yang Hospital, Capital Medical University, January 2018 to December 2018) and enrolled in the study with a mean age of 47 ± 14 years old, including 39 males and 16 females. The patients were divided into two groups by Glasgow Coma Scale score: the conscious disturbance group (GCS ≤ 14) and the non-conscious disturbance group (GCS>14). 32 patients were in the conscious disturbance group, including 27 males and 5 females. 22 cases were divided into the non-conscious disturbance group, including 22 males and 9 females. In addition, 11 healthy individuals were selected as the normal control group, including 6 males and 5 females. In the normal control group, the subjects aged from 18 to 63 years old (mean age,51 ± 14 years old).

Inclusion criteria: aged between 18 and 70 years old; clinically diagnosed with acute brain injury based on medical history, clinical symptoms and neurological testing; within 1 month after onset; the changes of consciousness and cognitive function approved by corresponding neurological testing; no hyperbaric oxygen therapy was performed after the onset. Exclusion criteria: a history of neurological diseases; with hepatic and renal insufficiency, multiple myeloma, autoimmune system diseases, blood system diseases, hypertension, heart disease, diabetes and other basic diseases; patients with severe mental diseases who couldn’t cooperate with examination and scoring; hyperbaric oxygen therapy was performed after onset.

### Hyperbaric oxygen therapy

When the condition of brain injury was stable, HBO therapy was performed as early as possible. And before HBO therapy, all patients were evaluated by past medical history and physical examination, chest computed tomography (CT), EKG and otoscope examination to ascertain the safety for HBO therapy. HBO treatment pressure was 2.0ATA, with pressure increase for 25 min, stabilization of the pressure and oxygen uptake for 60 min, and pressure decrease for 30 min. HBO therapy was once per day, and continuous treatment for 10 days as one session. Each session has an interval of 1 week.

### Examination

β2MG, β2MU, serum creatinine and urea nitrogen samples were detected respectively before hyperbaric oxygen therapy, after the first course of hyperbaric oxygen therapy and the second course of hyperbaric oxygen therapy.

In the early morning, the samples of venous blood and urine were taken on an empty stomach. The blood samples were centrifuged (3000 r/min) for 10 minutes. If there were floating objects in the urine samples, the supernatant was taken for detection after centrifugation. The obtained samples were detected by automatic biochemical analyzer of Beckman coulter AU5800. The concentrations of β2MG and β2MU were assessed with a kit (Shanghai Yingke Medical Biology Co., Ltd., Shanghai, China) by the method of latex enhanced turbidimetric immunoassay. The reference range of β2MG’s concentration was 0.8 ~ 1.8 mg/L, and that of β2MU was < 0.2 mg/L. The concentrations of serum creatinine and blood urea nitrogen were detected with the kit (Beijing Jiuqiang Biotechnology Co., Ltd., Beijing, China). The whole procedure was operated strictly according to the instructions by experienced laboratory physicians.

### Clinical evaluation

Consciousness and cognition testing were performed respectively before hyperbaric oxygen therapy, after the first course of hyperbaric oxygen therapy and the second course of hyperbaric oxygen therapy. These tests included Glasgow Coma Scale (GCS), Coma Recovery Scale-Revised score (CRS-R), Mini-Mental State Examination (MMSE) and Montreal Cognitive Assessment Scale (MoCA). Consciousness and cognitive status of the enrolled patients were assessed by the same physician who learned and mastered these tests. GCS and CRS-R scores were used in the conscious disturbance group, while MMSE and MoCA scores were used in the non-conscious disturbance group.

### Statistical analysis

The statistical analysis was performed using SPSS 24.0 (SPSS IBM Corp., Armonk, NY, USA). The measurement data was expressed by means ± standard deviation (SD). For data with a normal distribution, the independent-samples t-test was used for comparisons among groups. The enumeration data are expressed as a percentage. One-way repeated measures analysis of variance (one-way repeated measures ANOVA) was used for intra-group comparisons. The linear data of β2MG and score grade was analyzed by Spearman’s rank correlation analysis. And using the receiver operating characteristic curve (ROC curve) to analyze the relationship between β2MG and disturbance of consciousness. Statistical significance is defined as *p* < 0.05.

## Results

### General clinical characteristics

A total of 54 Chinese patients with brain injury were admitted to hospital (Beijing Chao-Yang Hospital, Capital Medical University, January 2018 to December 2018) and enrolled in the study with a mean age of 47 ± 14 years old, including 39 males and 16 females. According to clinical symptoms and GCS scores, the patients were divided into the conscious disturbance group and the non-conscious disturbance group, of which 32 were in the conscious disturbance group and 22 in the non-conscious disturbance group.

In the enrolled patients, 19 patients had no history of surgical intervention after brain injury, 26 patients received surgery within one day from the onset of brain injury, 6 patients received surgery on day 2, and 3 patients received surgery on day 3 or 4. The operative time among the different consciousness groups was significantly different (*P* = 0.009).

Among the etiologies of brain injury, 31 patients belonged to spontaneous cerebral hemorrhage, including 13 in basal ganglia, 2 in brain stem, 2 in cerebellum, 3 in frontal lobe, temporal lobe and parietal lobe, and 11 in other sites. There were 10 cases of subarachnoid hemorrhage, 7 cases of cerebral contusion and laceration, 3 cases of extradural hemorrhage, 1 case of subdural hemorrhage and 1 case of ruptured aneurysm. Among the different consciousness groups, there were significant differences in spontaneous cerebral hemorrhage and cerebral contusion and laceration (*p* < 0.050), but there was no significant difference in other etiologies.

The clinical symptoms of patients with brain injury mainly include conscious disturbance, dizziness and headache, nausea and vomiting, transient loss of consciousness, limb weakness, urinary incontinence and other symptoms (inarticulation, visual field defect, and blurred vision). According to the results of statistical analysis, the clinical symptoms of this study were significant differences in conscious disturbance, urinary incontinence, transient loss of consciousness and visual impairment between the conscious disturbance group and the non-conscious disturbance group (*p* < 0.050), and there was no significant difference in other symptoms. (Table 1).

**Table 1:** Baseline characteristics

	Brain injury (*n* = 54)	Consciousness disorder (*n* = 32)	Non-consciousness disorder (*n* = 22)	*P* value
Median age (years)	47 ± 14	52 ± 14	50 ± 13	0.734
Gender (male = n%)	39 (72%)	27 (84%)	13 (59%)	0.052
Time from onset to surgery				0.009
No surgery	19	5	14	
Day 0–1	26	22	4	
Day 2	6	4	2	
Day 3–4	3	1	2	
Etiologies of brain injury				
Spontaneous intracerebral hemorrhage	31	23	8	0.010
Basal ganglia	13	11	2	0.260
Brain stem	2	0	2	0.013
Cerebellum	2	2	0	0.389
Frontal Lobe; Parietal Lobe; Temporal Lobe	3	2	1	0.754
Other	11	8	3	0.890
Subarachnoid hemorrhage	10	4	6	0.170
Cerebral contusion and laceration	7	1	6	0.009
Extradural hemorrhage	3	2	1	0.788
Subdural hemorrhage	2	1	1	0.786
Intracranial aneurysm	1	1	0	0.403
Clinical symptoms				
Conscious disturbance	32	32	0	0.000
Headache and dizziness	17	7	7	0.413
Nausea and vomiting	16	10	6	0.753
Transient loss of consciousness	8	0	8	0.000
Limb weakness	8	5	3	0.840
Urinary incontinence	4	4	0	0.000
Others (inarticulate, defect of visual field, blurred vision)	6	1	5	0.024
GCS score on admission				
3–8		24		
9–12		7		
13–15		1		
MMSE score on admission				
0–9			0	
10–20			5	
21–27			17	

Continuous variables are expressed as percentage or mean ± standard deviation, and categorical variable are expressed in terms of n (%)

*GCS* Glasgow coma scale, *MMSE* Mini-mental State Examination

As shown in Table 2, there was no significant difference in the time of onset to HBOT and Marshall CT classification between the conscious disturbance group and the non-conscious disturbance group. However, the results showed that the hempill score in the conscious disturbance group was higher than that in the non-conscious disturbance group, and the difference was statistically significant.

**Table 2:** Imaging and starting time for HBOT

	Brain injury (*n* = 54)	Conscious disturbance (*n* = 32)	Non-conscious disturbance (*n* = 22)	*P* value
Time from onset to HBOT				0.065
Day 0–14	4	1	3	
Day 15–21	18	9	9	
Day **≥** 22	32	22	10	
Hempill score				0.017
0–1		1	4	
2		3	3	
3		3	1	
4		12	1	
**≥** 5		3	1	
Marshall CT classification				0.140
1		2	7	
2		1	0	
3		2	0	
4		0	2	
5 or 6		5	2	

*HBOT* Hyperbaric oxygen therapy

### Comparison of clinical data between brain injury group and control group

Comparing the general characteristics of the brain injury group and the normal control group, the results showed that there was no significant difference in age and gender between the brain injury group and the control group. The concentrations of β2-microglobulin in the brain injury group showed a downward trend at different time points, and there was a significant difference in β2MG before and after the first course of HBO therapy compared with the control group (*P* = 0.001, *P* = 0.002), while there was no significant difference after the second course of HBO therapy (*P* = 0.067). β2MU was significantly different from the control group before hyperbaric oxygen treatment (*P* = 0.006), and there was no significant difference from the control group after hyperbaric oxygen treatment (*P* = 0.258, *P* = 0.308). There was a significant difference in serum creatinine between the brain injury group and the control group (*P* < 0.05), but there was no significant difference in urea nitrogen compared with the control group (*P* > 0.05), as shown in Table 3.

**Table 3:** Comparison of clinical data between the brain injury and the control groups

Category	Brian injury group(*n* = 54)	Control group (*n* = 11)	*P*
Age	47 ± 14	51 ± 14	0.321
Gender (male = n%)	39 (72%)	6 (54%)	0.200
β2MG before HBOT	2.64 ± 0.79	1.73 ± 0.39	0.001
β2MU before HBOT	6.82 ± 20.2	0.17 ± 0.16	0.006
SCR before HBOT	52 ± 14	64 ± 11	0.016
BUN before HBOT	5.3 ± 2.2	4.3 ± 1.2	0.190
β2MG after 1st course HBOT	2.58 ± 0.76	1.73 ± 0.39	0.002
β2MU after 1st course HBOT	2.07 ± 7.46	0.17 ± 0.16	0.258
SCR after 1st course HBOT	47 ± 16	64 ± 11	0.004
BUN after 1st course HBOT	4.4 ± 1.4	4.3 ± 1.2	0.907
β2MG after 2nd course HBOT	2.14 ± 0.66	1.73 ± 0.39	0.067
β2MU after 2nd course HBOT	0.65 ± 2.09	0.17 ± 0.16	0.308
SCR after 2nd course HBOT	47 ± 15	64 ± 11	0.004
BUN after 2nd course HBOT	4.1 ± 1.3	4.3 ± 1.2	0.715

Comparison of clinical data between the brain injury and the control groups. Continuous variables are expressed as percentage or mean ± standard deviation, categorical variables are showed as percentage(n%). *P* < 0.05 indicates statistical difference.β2MG, β2-microglobulin (mg/L); β2MU, urineβ2-microglobulin (mg/L); *SCR* Serum creatinine (μmol/L), *BUN* Blood urea nitrogen (μmol/L)

### Comparison of data between the conscious disturbance group and non-conscious disturbance group

There was no significant difference in age and sex between conscious disturbance group and non-conscious disturbance group (*P* > 0.05). The concentration of β2MG in the conscious disturbance group was significantly higher than that in the non-conscious disturbance group before HBOT, after the first and the second course of HBOT, and the difference was statistically significant (*P* < 0.05). The concentration of β2MU in the conscious disturbance group was significantly higher than that in the non-conscious disturbance group only before HBOT, and the difference was statistically significant (*P* < 0.05), but there was no significant difference between the first and the second courses of HBOT (*P* > 0.05). There was no significant difference between serum creatinine and blood urea nitrogen before and after HBOT (Table 4.)

**Table 4:** Comparison of data between the conscious disturbance group and non-conscious disturbance group

Category	Conscious disturbance group(*n* = 32)	Non-conscious disturbance group(*n* = 22)	*P*
Age	52 ± 14	50 ± 13	0.734
Gender (male = n%)	27 (84%)	13 (59%)	0.052
β2MG before HBOT	2.89 ± 0.88	2.20 ± 0.96	0.009
β2MU before HBOT	10.78 ± 20.2	1.76 ± 7.02	0.035
SCR before HBOT	52 ± 15	53 ± 14	0.878
BUN before HBOT	5.6 ± 2.5	4.7 ± 1.5	0.208
β2MG after 1st course HBOT	2.77 ± 0.65	2.02 ± 0.49	0.029
β2MU after 1st course HBOT	3.36 ± 9.03	0.28 ± 0.28	0.384
SCR after 1st course HBOT	47 ± 17	47 ± 13	0.978
BUN after 1st course HBOT	4.3 ± 1.2	4.5 ± 2.1	0.848
β2MG after 2nd course HBOT	2.59 ± 0.76	1.79 ± 0.35	0.025
β2MU after 2nd course HBOT	1.15 ± 2.64	0.13 ± 0.89	0.366
SCR after 2nd course HBOT	48 ± 15	45 ± 18	0.783
BUN after 2nd course HBOT	4.2 ± 1.5	4.0 ± 1.2	0.868

Continuous variables are expressed as percentage or mean ± standard deviation, categorical variables are showed as percentage(n%). *P* < 0.05 indicates statistical difference. β2MG, β2-microglobulin (mg/L); β2MU, urine β2-microglobulin (mg/L); *SCR* Serum creatinine (μmol/L), *BUN* Blood urea nitrogen (μmol/L)

### Analysis of the variation tendency of β2MG and β2MU’s concentrations

The method of One-way Repeated Measures ANOVA was used to analyze the variation tendency of β2MG and β2MU’s concentrations within each group and between groups before hyperbaric oxygen therapy, after the first and second course of hyperbaric oxygen therapy. Through the Mauchly’s test of sphericity, the results showed that the concentrations of β2MG satisfied the test (W = 0.881, *P* = 0.091). Therefore, at different detection time points, the intra-group comparison of β2MG’s concentrations had significant differences (*P* = 0.000), while the comparison among groups of β2MG’s concentrations was no significant difference (*P* = 0.929).

**Fig. 1:**
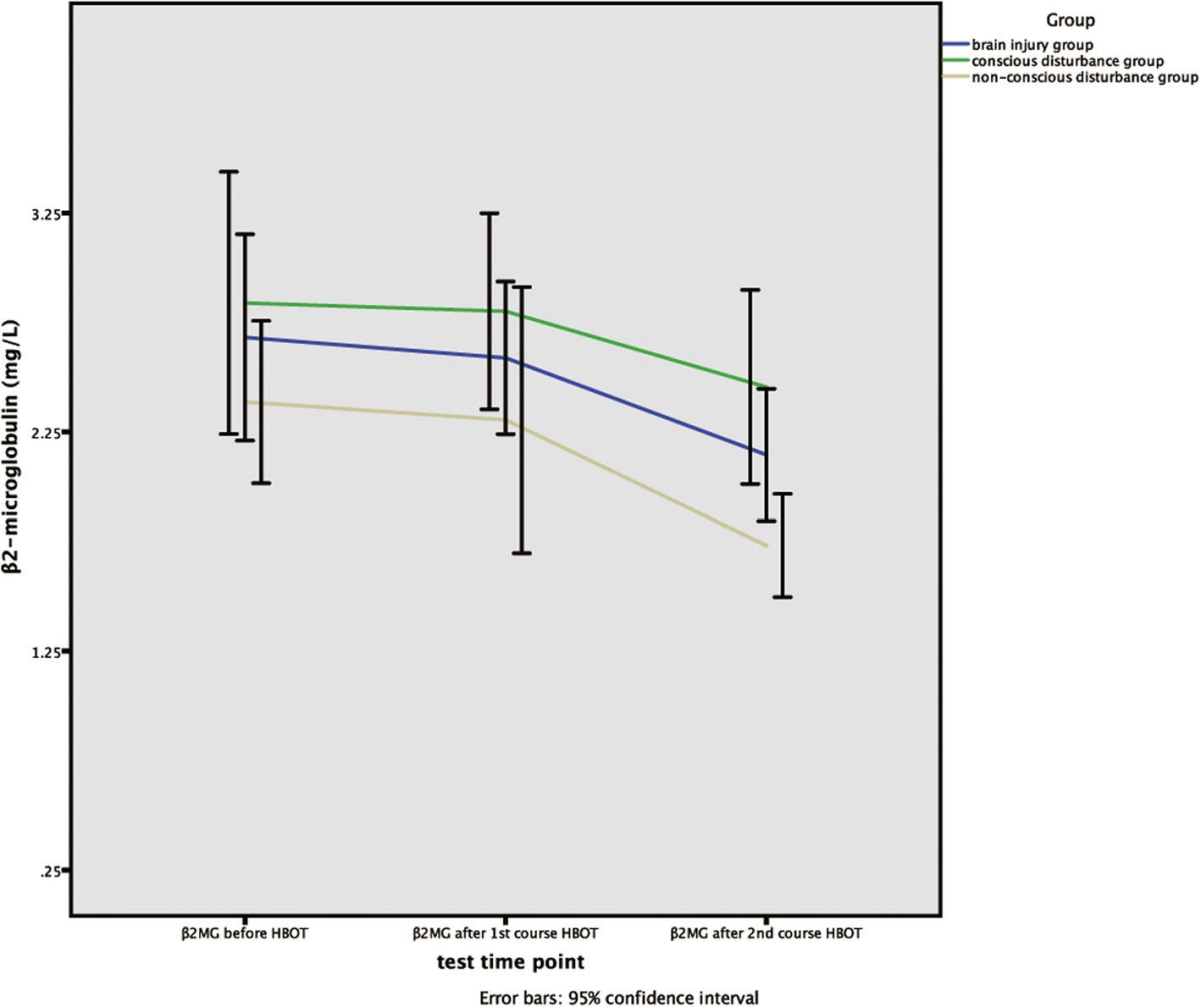
The variation tendency of β2MG’s concentrations in brain injury, consciousness disturbance and non-conscious disturbance group. X-axis indicates the particular experimental time points, which respectively are before hyperbaric oxygen therapy, after the first course of hyperbaric oxygen therapy, and after the second course of hyperbaric oxygen therapy. Y-axis is used to represent concentrations (mg/L). β2MG, β2-microglobulin, HBOT, Hyperbaric oxygen therapy

**Fig. 2:**
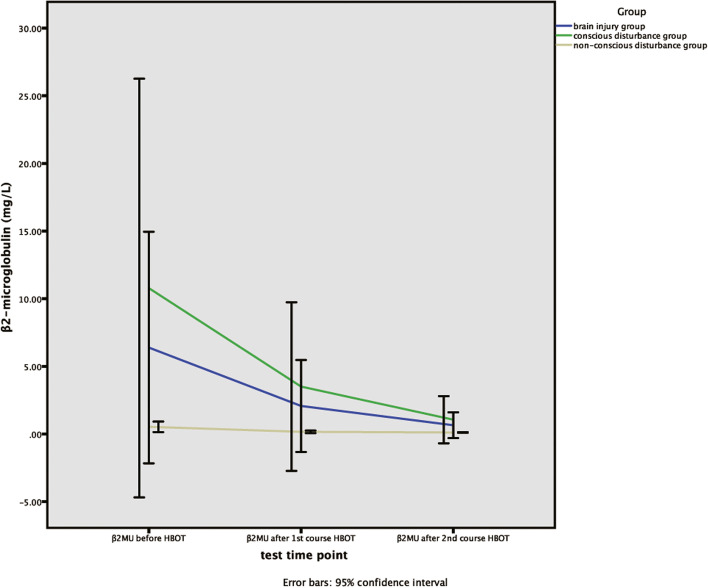
The variation tendency of β2MU’s concentrations in brain injury, consciousness disturbance and non-conscious disturbance group. X-axis indicates the particular experimental time points, which respectively are before hyperbaric oxygen therapy, after the first course of hyperbaric oxygen therapy, and after the second course of hyperbaric oxygen therapy. Y-axis is used to represent concentrations (mg/L). β2MU，urine β2-microglobulin, HBOT, Hyperbaric oxygen therapy

Through the Mauchly’s test of sphericity, the results showed that the concentrations of β2MG were not in accordance with the sphericity test(W = 0.181,*P* = 0.000). The method of multivariate statistical analysis was used to analyze β2MU’s concentration at different time points, the results demonstrated that there was no significant difference within group (*P* = 0.059), and there was no significant difference among the groups (*P* = 0.260), but the overall variation tendency of β2MU was decreasing. (Table 5, Figs. 1-2).

**Table 5:** The variance analysis of repeated measurements for β2MG and β2MU

	Time point	Group			Machly W	***P***_**1**_	F		***P***_**2**_	
		Brain injury group	Conscious disturbance group	Non-conscious disturbance group			Time	Time*group	Time	Time*group
β2MG	Before HBOT After 1st course After 2nd course	2.64 ± 0.79 2.58 ± 0.76 2.14 ± 0.66	2.89 ± 0.88 2.77 ± 0.65 2.59 ± 0.76	2.38 ± 0.48 2.30 ± 0.79 1.73 ± 0.30	0.881	0.091	10.251	0.189	0.000	0.929
β2MU	Before HBOT After 1st course After 2nd course	6.82 ± 20.2 2.07 ± 7.46 0.65 ± 2.09	10.78 ± 20.2 3.36 ± 9.03 1.15 ± 2.64	0.52 ± 0.50 0.15 ± 0.12 0.11 ± 0.03	0.181	0.000	38.000	39.000	0.059	0.026

Continuous variables are expressed as percentage or mean ± standard deviation, and *P* < 0.05 indicates statistical difference. *P*1: the *p*-value for the sphericity test. *P*2 of β2MG: *p*-value for the within-subject effect test. *P*2 of β2MU: *p*-value for the multivariate test. β2MG: β2-microglobulin, β2MU: urine β2-microglobulin, *HBOT* Hyperbaric oxygen therapy

### Analysis of the variation tendency of serum creatinine and blood urea nitrogen’s concentrations

**Fig. 3:**
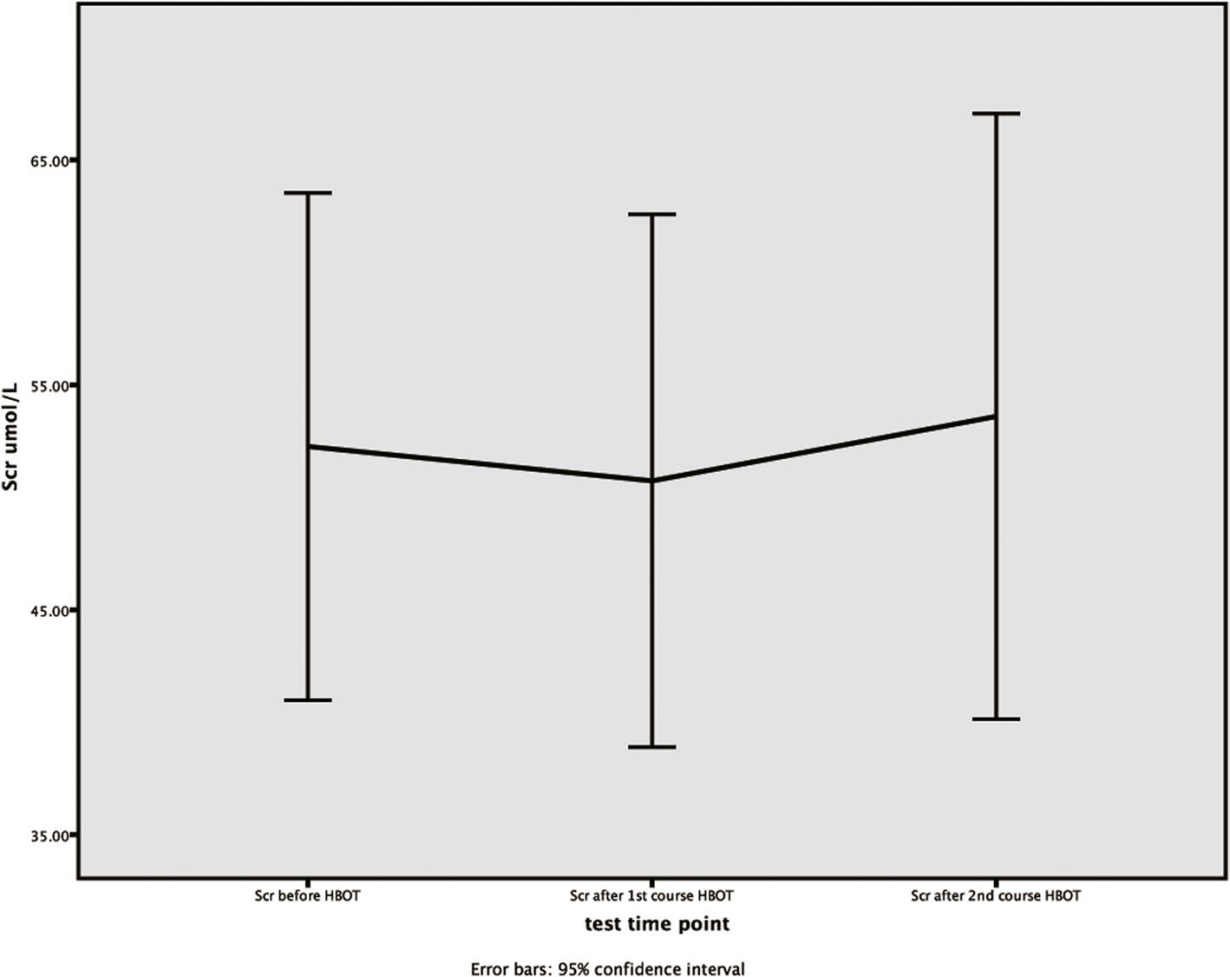
The variation tendency of serum creatinine’s concentrations in brain injury group. X-axis indicates the particular experimental time points, which respectively are before hyperbaric oxygen therapy, after the first course of hyperbaric oxygen therapy, and after the second course of hyperbaric oxygen therapy. Y-axis is used to represent concentrations (μmol/L). Scr, serum creatinine

**Fig. 4:**
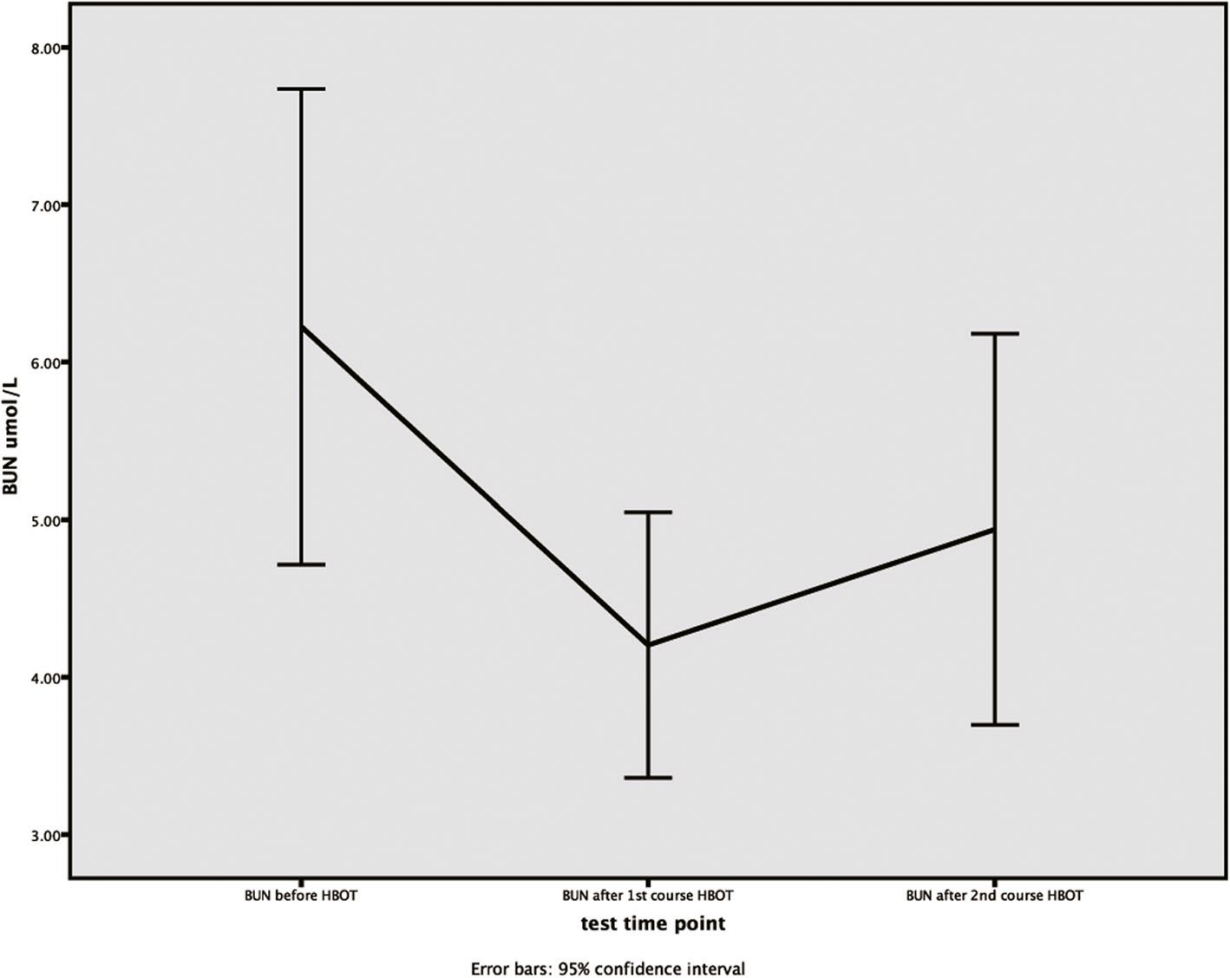
The variation tendency of BUN’s concentrations in brain injury group. X-axis indicates the particular experimental time points, which respectively are before hyperbaric oxygen therapy, after the first course of hyperbaric oxygen therapy, and after the second course of hyperbaric oxygen therapy. Y-axis is used to represent concentrations (μmol/L). BUN, blood urea nitrogen

The method of One-way Repeated Measures ANOVA was used to analyze the variation tendency of serum creatinine and blood urea nitrogen’s concentrations in brain injury group before hyperbaric oxygen therapy, after the first and second course of hyperbaric oxygen therapy. Although the concentrations of serum creatinine in brain injury group met the spherical test (W = 0.589, *P* = 0.054) after Mauchly’s spherical hypothesis test, there was no significant difference within the groups at different time points (*P* = 0.477). The concentrations of serum creatinine in brain injury group met the spherical test (W = 0.647, *P* = 0.092), and there was significant difference within the groups at different time points (*P* = 0.035). (Table 6, Figs. 3-4).

**Table 6:** The variance analysis of repeated measurements for SCR and BUN

Variable	Before HBOT	After 1st course of HBOT	After 2nd course of HBOT	Machly W	*P*1	F	*P*2
SCR	52 ± 14	47 ± 17	48 ± 15	0.589	0.054	0.665	0.477
BUN	5.3 ± 2.2	4.3 ± 1.2	4.2 ± 1.5	0.647	0.092	4.538	0.035

continuous variables are expressed as percentage or mean ± standard deviation, and *P* < 0.05 indicates statistical difference. *P*1: the *p*-value for the sphericity test. *P*2: *p*-value for the within-subject effect test

*SCR* Serum creatinine (μmol/L), *BUN* Blood urea nitrogen (μmol/L), *HBOT* Hyperbaric oxygen therapy

### Correlation analysis between consciousness, cognition score and β2-microglobulin’s concentration

#### To analyze whether the concentrations of β2MG and β2MU in the consciousness disorder group are correlated with GCS score, GCS score grade, and CRS-R score

According to Spearman’s rank correlation analysis, the result suggested that β2MU’s level was positively correlated with GCS score grade (*R* = 0.354, *P* = 0.019), negatively correlated with GCS score (*R* = -0.351, *P* = 0.019), and negatively correlated with CRS-R score (*R* = -0.438, *P* = 0.003). There was no significant correlation between β2MG’s level and GCS score grade, GCS score and CRS-R score (*P* = 0.931, *P* = 0.810, *P* = 0.268). That was, The lower the GCS score, the more severe the disturbance of consciousness and the higher the level of β2MU.

#### To analyze whether the levels of β2MG and β2MU in the non-consciousness disorder group are correlated with MMSE score, MMSE score grade, and MoCA score

According to Spearman’s rank correlation analysis, it was found that β2MG’s level was positively correlated with MMSE score grade (*R* = 0.598, *P* = 0.011), but had no significant correlation with MMSE score and MoCA score (*P* = 0.055, *P* = 0.119). There was no significant correlation between β2MU’s level and MMSE score, MMSE score and MoCA score (*P* = 0.390, *P* = 0.090, *P* = 0.715). That was, the more severe the cognitive impairment, the lower the MMSE score grade and the higher the β2MG level.

**Fig. 5:**
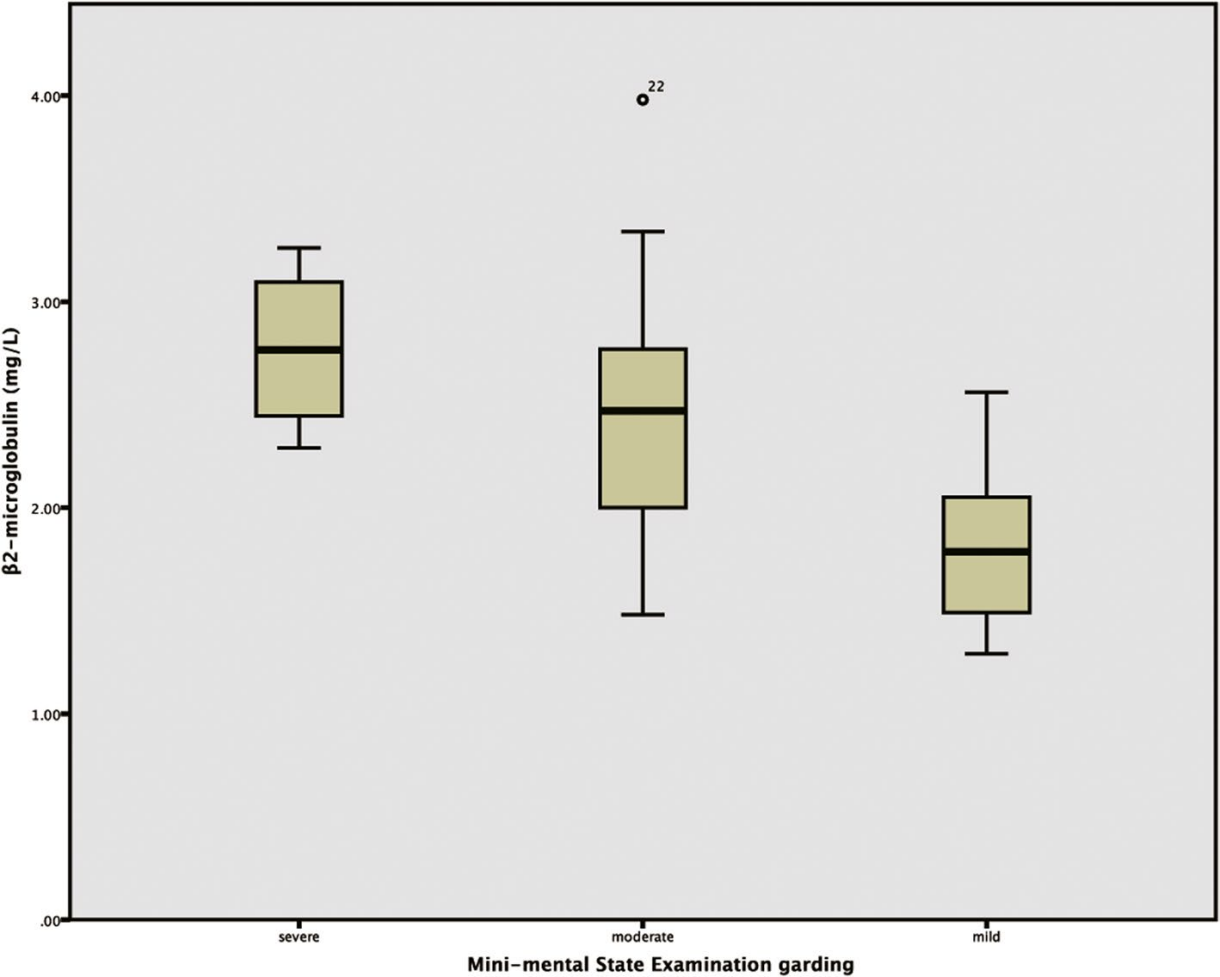
The average concentrations of β2MG in different MMSE scores in the non-conscious disturbance group. The X-axis is the score classification according to MMSE scale, which are mild, moderate, and severe, respectively, and the Y-axis is the concentrations of β2MG (mg/L)

Through the method of One-way ANOVA, the difference of β2MG concentrations was compared among the mild, moderate, severe grades of MMSE score. The results indicated that the concentrations of β2MG conformed to the normal distribution, and the homogeneity of variance test showed that the variance was homogeneous, and there was a significant difference in the concentration of β2MG among the different grades of MMSE score(*P* = 0.001). Comparisons between different grades indicated that there was a significant difference in the concentration of β2MG between mild grades (1.81 ± 0.35 mg/L) and moderate grades (2.51 ± 0.67 mg/L). In addition, compared the concentration of β2MG between mild and severe grades (2.77 ± 0.41 mg/L), the difference was statistically significant. Correspondingly, the *p* value was 0.003 and 0.002. However, there was no significant difference compared the concentration of β2MG between moderate and severe grades (*P* = 0.404). (Fig. 5).

#### Receiver operating characteristic curve (ROC curve) was used to evaluate the value of β2MG and β2MU’s levels in the evaluation of patients’ consciousness disorder

**Fig. 6:**
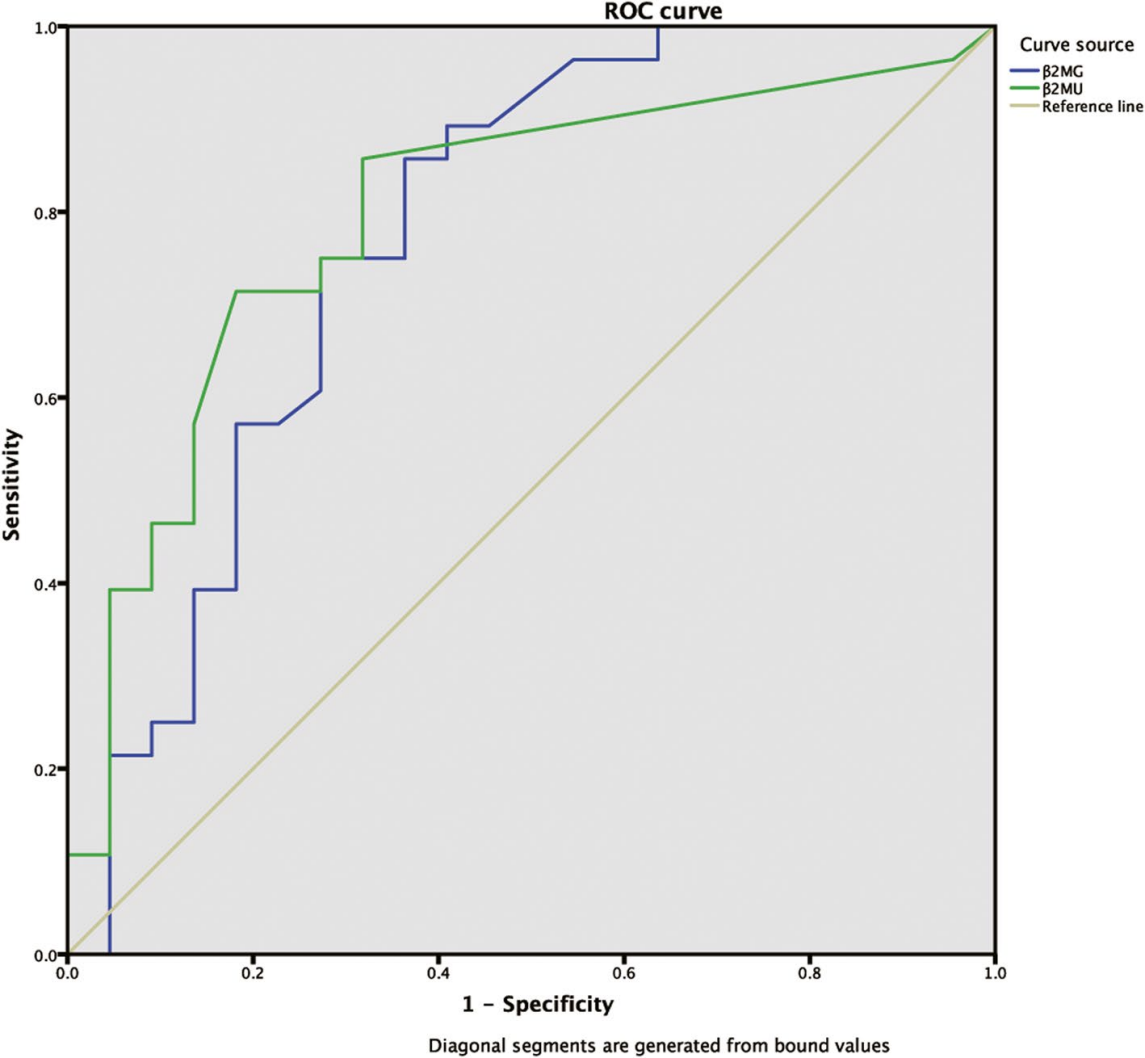
ROC analysis of consciousness disorder (GCS ≤ 14) in all patients in the consciousness disorder group. ROC, receiver operating characteristic. GCS, Glasgow coma scale

The degree of coma was evaluated by Glasgow Coma Scale (GCS). GCS > 14 is defined as clear consciousness, while GCS ≤ 14 is defined as impaired consciousness. The receiver operating characteristic curve (ROC curve) was used to assess the evaluation value of β2MG and β2MU’s levels for patients with impaired consciousness. The area under the curve (AUC) of β2MG was 0.775 (95%CI = 0.636 ~ 0.914, *P* = 0.001,cutoff = 2.15 mg/L, Sensitivity = 0.857,specificity = 0.636, and Youden index = 0.493). The AUC of β2MU was 0.796 (95% CI = 0.667 ~ 0.925, *P* = 0.000, cutoff =0.18 mg/L,sensitivity = 0.857, specificity = 0.682, and Youden index =0.539). As shown in Fig. 6.

### Discussion

β2-microglobulin is a small molecule protein produced by lymphocytes, platelets, and multinucleated leukocytes, existing on the membrane of all nucleated cells except red blood cells and placental trophoblast cells. It is produced and released at a constant rate. With the characteristics of freely passing through the glomerular filtration membrane, not being secreted by the renal tubules and affected by muscle metabolism and tubular secretions, β2-microglobulin is regarded as an ideal serum marker for evaluating the glomerular filtration rate. When the concentration of β2MG increases, it indicates that the glomerular filtrating function is impaired or the filtration load increases. Various primary or secondary diseases, including kidney diseases, autoimmune diseases, hematological diseases, multiple myeloma and so on, can increase the concentration of β2MG. In recent years, it has been found that β2MG is also correlated with the occurrence of cardiovascular and cerebrovascular diseases. β2MG not only participates in the pathophysiological process of cardiovascular and cerebrovascular diseases, but also has a certain effect on the severity and prognosis of these diseases. The results of this study also confirmed the correlation between β2MG and brain injury.

### Correlation between β2-microglobulin and brain injury

In this research, it was found that the content of β2MG in patients with brain injury was higher than that in the normal control group, and the content of β2MG in the conscious disturbance group was higher than that in the non-conscious disturbance group. And there were significant differences in results. These suggested that the concentration of β2MG was positively correlated with the degree of brain injury. When brain injury occurs, ischemia and hypoxia of brain cells lead to necrosis of brain tissue, and a large number of white blood cells accumulate in the necrotic area, triggering the acute inflammatory reactions characterized by inflammatory response, the production of anti-inflammatory factors, and neutrophil aggregation. The increase of neutrophil can upregulate the synthesis and secretion of β2MG. The enhancement of inflammatory response suggests that the immune system was under the state of stress, as well as the secretion and release of neurotransmitters and hormones will change. The neuroendocrine system may affect and regulate the immune system, increase the number and activity of lymphocytes, and promote the production and release of β2MG. And the content of β2MG increase with the severity of the disease. In addition, the renin-angiotensin-aldosterone system (RAAS) in the body is activated after brain injury, which stimulates the release of renin and angiotensin II, causing vasoconstriction of brain and kidney, aggravating cerebral ischemia and hypoxia damage, and reducing glomerular filtration rate, leading to the increase of β2MG in vivo [13]. Therefore, when brain injury occurs, it can be observed that the content of β2-microglobulin in patients with brain injury is significantly higher than that of the normal control group, and the content of β2-microglobulin is positively correlated with the severity of brain injury. What’s more, the content of β2-microglobulin in the conscious disturbance group with severe brain injury was higher than that in the non-conscious disturbance group. After hyperbaric oxygen therapy, the content of β2-microglobulin decreased obviously in the brain injury group, and there was a significant difference at different time points in the intra-group comparison of β2MG. But there was no significant difference in the content of β2MG after two courses of hyperbaric oxygen therapy compared with the control group. Hyperbaric oxygen therapy can increase the oxygen content of brain tissue and the diffusion distance of oxygen in blood, restore the aerobic metabolism of brain cells, improve the ischemic hypoxia state of brain tissue, break the vicious circle of hypoxia-edema, improve the oxygen supply of brain cells in ischemic penumbra, and increase the activity of brain cells in this area. What’s more, hyperbaric oxygen can stabilize neutrophils, inhibit the infiltration and aggregation of inflammatory cells, and reduce inflammatory reactions. Hyperbaric oxygen can also regulate the function of immune system [14], reduce the production of immunoglobulin and inhibit the formation of immune complexes, improve the phagocytic function of macrophages and reduce local immune responses. Consequently, we think that β2MG can be used as a monitoring indicator of brain injury so as to judge the condition of the disease in the early stage, and hyperbaric oxygen treatment can effectively alleviate the body’s immune inflammatory response and reduce the serum content of β2MG.

In this study, it was explored that the concentration of β2MU in the brain injury group was higher than that in the control group before hyperbaric oxygen therapy, and the concentration of β2MU was correlated with the degree of brain injury. Under the physiological conditions, β2-microglobulin is reabsorbed in renal tubules, and the content of β2-microglobulin in urine is extremely small. So only a small amount of β2-microglobulin is excreted with urine. When the content of β2-microglobulin increased after brain injury, its glomerular filtration increased and exceeded the reabsorption capacity of renal tubule so that the excretion of β2-microglobulin increased with urine. Meanwhile, acute kidney injury, caused by the excitement of sympathetic nerve, the release of inflammatory factors, and the stress state of the immune system and so on, can affect the glomerular filtration rate and renal tubular blood flow volume. Renal tubular epithelial cells have suffered ischemia and hypoxia due to the reduction of renal tubular blood flow volume and functional impairment, resulting in the reabsorption disorder of β2-microglobulin and increased excretion from urine. In this study, β2MU showed a downward trend after hyperbaric oxygen therapy, but there was no significant difference within the group at different time points, and there was no significant difference between the hyperbaric oxygen treatment group and the normal control group. First of all, hyperbaric oxygen therapy can increase tissue oxygen partial pressure, correct cellular ischemia and hypoxia, adjust water and electrolyte balance, improve local blood flow in the kidney, and increase glomerular filtration rate. Secondly, after admission, the use of vasoactive drugs, mannitol or diuretics and other drugs affected the glomerular filtration rate and renal tubular reabsorption function. The above multiple factors may affect the detection results of β2MU, so that there is no significant difference in the comparison of β2MU between and within groups after hyperbaric oxygen treatment. It is hoped that the meaningful results can be obtained by expanding the sample size, monitoring glomerular filtration rate and comprehensive treatment to evaluate the content of β2MU in patients with brain injury in the future.

### Correlation of β2-microglobulin with consciousness and cognitive impairment

In this study, when the blood and urine samples of all enrolled patients were collected, the consciousness and cognition functions were evaluated. The results showed that there was a certain correlation between the concentration of β2-microglobulin and the functions of consciousness and cognition. Through the method of Spearman’s rank correlation analysis to analyze the correlation between the concentrations of β2MG and β2MU and the scores of consciousness and cognition, the results showed that the concentration of β2MU was negatively correlated with GCS score in the patients with consciousness impairment. The concentration of β2MG in non-conscious disturbance group was negatively correlated with the MMSE score. Li Zhi-Guo [3], Liu Yang [15] et al., also found that different degrees of brain injury and neurological impairment were related to the concentration of β2MG in blood. The larger the lesion of brain injury and the more severe the neurological deficit, the higher the concentration of β2MG in patients and the higher the content of β2-microglobulin excreted by the kidneys. β2-microglobulin is secreted by lymphocytes, neutrophils, mononuclear macrophages, etc. After the occurrence of brain injury, the immune system in the body produces macrophages to phagocytic pathogens and apoptotic cells, inducing peripheral neutrophils, monocytes and lymphocytes to secrete β2-microglobulin. Furthermore, β2-microglobulin may also be involved in mediating inflammatory response as an inflammatory mediator. However, the excessive effects of immune response and inflammatory factors can lead to neuronal damage due to cell dysfunction and oxidative stress [16]. In this research, it explored that the concentration of β2MU in patients was correlated with disturbance of consciousness. Maybe it attributed to the similar anatomical structure and functional characteristics of renal vessels and cerebrovascular vessels [17]. Both of them need to maintain stable and continuous high blood perfusion in a low vascular resistance system, so they have common risk factors. When factors resulting in microvascular damage have an effect on kidney, such as inflammatory factors and immune factors after brain injury, it leads to direct or indirect glomerular damage, the change of glomerular filtration rate, and destruction of renal tubular epithelium function. It also leads to filtration barrier damage and reabsorption dysfunction, increase the excretion of β2MU. And the concentration of β2MG and β2MU increase with the aggravation of brain injury. Therefore, the results of this study indicated that the β2MU’s concentration of patients in the impaired consciousness group maybe reflect the severity of the brain injury. Studies on β2-microglobulin and cognitive impairment found that the concentration of β2-microglobulin was positively correlated with the degree of cognitive impairment [8–10, 18]. High concentrations of β2-microglobulin are characterized in diseases with a high incidence of cognitive impairment, such as chronic kidney disease, multiple myeloma, and central nervous system infections. Age-related cognitive impairment occurs when the production of β2-microglobulin is excessive and the clearance of β2-microglobulin is insufficient. This may be associated with the immunological and neuroregulatory functions of β2-microglobulin, which alter brain development and cognitive function by modulating neural regeneration and synaptic plasticity behavior. The process of major histocompatibility complex-dependent immune responses in the brain may damage neurons, and β2-microglobulin is involved in this process [19–21]. β2-microglobulin can also act on hippocampal neural progenitor cells (NPC) to affect their self-renewal, proliferation and neuronal differentiation, leading to cognitive deficits. In the research on the correlation between β2-microglobulin and acute changes in cognitive impairment [22], in acute and stable environments, there is a significant correlation between the concentrations of β2-microglobulin and cognitive function, and this phenomenon is reversible. Although the correlation is not equal to causality, β2-microglobulin may be a systemic stimulator that impairs cognitive function and neurogenesis. As a result, in this study, it was found that the concentration of β2MG was positively correlated with the degree of cognitive impairment through the evaluation of MMSE score in the non-conscious disturbance group, and moreover, the concentration of β2MG was significantly different between mild cognitive impairment and moderate to severe cognitive impairment. Thus, it demonstrated that the change of β2MG’s concentration could make a preliminary judgment on the improvement of cognitive state and prognosis of the non-consciousness disorder patients.

Through further analysis of the receiver operating characteristic curve to study the value of β2-microglobulin in judging whether the patient has a disorder of consciousness, it is found that the concentrations of β2MG and β2MU in patients with brain injury are correlated with conscious disturbance. Consequently, the results of this study are helpful for early clinical diagnosis and timely intervention, provide guidance for the formulation of treatment proposals, and make a preliminary judgment on the development and prognosis of patients by monitoring the changes of β2-microglobulin in order to guide clinical treatment.

To sum up, the content of β2-microglobulin in patients with brain injury was significantly increased, and was positively correlated with the severity of brain injury, consciousness disturbance and cognitive impairment. By detecting the change trend of β2-microglobulin, it is possible to understand the changes in the patient’s condition and make a preliminary judgment on the prognosis of the condition. Hyperbaric oxygen as a treatment method can promote the recovery of consciousness in patients with brain injury, enhance cognitive function, and improve the condition and prognosis of the disease. However, there are still some shortcomings in this study. First of all, the sample size is too small. In the future, expanding the clinical sample size can further study the correlation between β2-microglobulin and brain injury, and at the same time, the renal function of patients with brain injury can be better assessed by calculating the glomerular filtration rate. Secondly, the content and change trend of β2-microglobulin in patients with brain injury without hyperbaric oxygen therapy can be detected simultaneously in the study, and more meaningful findings are expected through comparative observation.

## Summary and conclusion

In conclusion, blood β2-microglobulin，which was significantly increased in patients with brain injury and positively correlated with the degree of brain injury and cognitive impairment, can be considered as a monitoring index to indicate and assess the condition at an early stage. The content of urine β2-microglobulin increased early in patients with brain injury and disturbance of consciousness, which was positively correlated with the degree of consciousness disorders. The variation tendency of β2-microglobulin can reflect patients’ consciousness and cognitive degree, and provide a relevant basis for preliminary judgment of prognosis of patients.

## Data Availability

The additional unpublished data can be available by the mail of the corresponding author.

## Abbreviations

GCS: Glasgow Coma Scale
β2MG: β2-microglobulin
β2MU: urine β2-microglobulin
MMSE: Mini-Mental State Examination
ROC curve: Receiver operating characteristic curve
AUC: Area under the curve
MHC-I: Major histocompatibility complex I
NPC: Neural progenitor cell
CRS-R score: Coma Recovery Scale-Revised score
MoCA: Montreal Cognitive Assessment Scale
ANOVA: analysis of variance
HBOT: Hyperbaric oxygen therapy
SCR: serum creatinine
BUN: Blood urea nitrogen
RAAS: Renin-angiotensin-aldosterone system
